# Do Gastrointestinal Symptoms Affect the Endoscopic Outcome in Anemic Premenopausal Women Due to Iron Deficiency: A Multicenter Study From Basrah-Iraq

**DOI:** 10.7759/cureus.14524

**Published:** 2021-04-16

**Authors:** Samih A Odhaib, Miaad J Mohammed, Saad S Hammadi

**Affiliations:** 1 Adult Endocrinology, Faiha Specialized Diabetes, Endocrine and Metabolism Center (FDEMC) College of Medicine, University of Basrah, Basrah, IRQ; 2 Diagnostic Radiology, Al-Refaee General Hospital, Thi-Qar Health Directorate, Thi-Qar, IRQ; 3 Internal Medicine, College of Medicine, University of Basrah, Basrah, IRQ

**Keywords:** anemia, endoscopy, iron deficiency, gastrointestinal, premenopausal, symptom

## Abstract

Background and objectives: The most common cause for iron deficiency anemia (IDA) in women before menopause is menstrual blood loss. The persistence of digestive symptoms despite iron supplementation is the only indication for gastrointestinal (GI) endoscopy in premenopausal women (PW) with IDA. We evaluated how the GI symptomatology manifestation affects the GI endoscopy diagnostic outcome in this cohort.

Materials and methods: This is an observational, multicenter retrospective evaluation of 245 PW admitted for GI endoscopic diagnosis for the etiology of IDA from 2006 to 2016. Baseline measurements included hemoglobin, iron status tests, and red blood corpuscle morphological evaluation. We evaluated the relationships of different endoscopic findings to the severity of IDA, different demographic characteristics, and hospitalization duration.

Results: The mean age was 40±7 years. The duration of hospitalization was neither associated with age nor the IDA severity. The IDA was mild to moderate. More than 53% (n=131) had either a negative study or nonspecific inflammatory changes. Around 16% (n=39) had GI malignancies. There was a significant association between initial GI symptoms with endoscopic GI finding and GI malignancy diagnosis in particular. The relationship loses its power during further assessment by general univariate analysis.

Conclusion: A considerable percentage of anemic PW due to iron deficiency has an endoscopically-diagnosed pathology for IDA determined during GI endoscopy. The GI symptoms' phenotypes were unrelated to the endoscopically-diagnosed GI lesion location, even if they were malignant. Therefore, the determination of IDA severity must be thoroughly and individually determined.

## Introduction

The commonest cause for iron deficiency anemia (IDA) in premenopausal women (PW) is menstrual blood loss [1], yet the increasing demand during pregnancy and breastfeeding, and dietary deficiency, may be contributing factors [1-3]. It is questionable to refer all PW to gastrointestinal (GI) endoscopic studies to investigate the etiology of IDA [1,4-5], and instead to reserve endoscopic evaluation for the PW with persistent symptoms, high risk for GI malignancy due to relevant strong family history, or those with treatment-resistant IDA [1,6].

The current guidelines recommend GI endoscopic screening of all PW with IDA for celiac disease (CD), asymptomatic PW with IDA [1], and in hysterectomized anemic PW [7].

The pathophysiology behind IDA in menstruating women does not rule out the coexistence of GI causes and menstrual blood loss as an etiology [5,8]. As a result, the clinical management is controversial and complex [8], and many PW with IDA are treated with oral iron supplements without symptom-directed GI endoscopic evaluation that may reveal significant GI lesions [5].

Our objective in this study is the retrospective evaluation of gastrointestinal endoscopic findings in premenopausal women with iron deficiency anemia, whatever the symptomatology.

## Materials and methods

**Figure 1:**
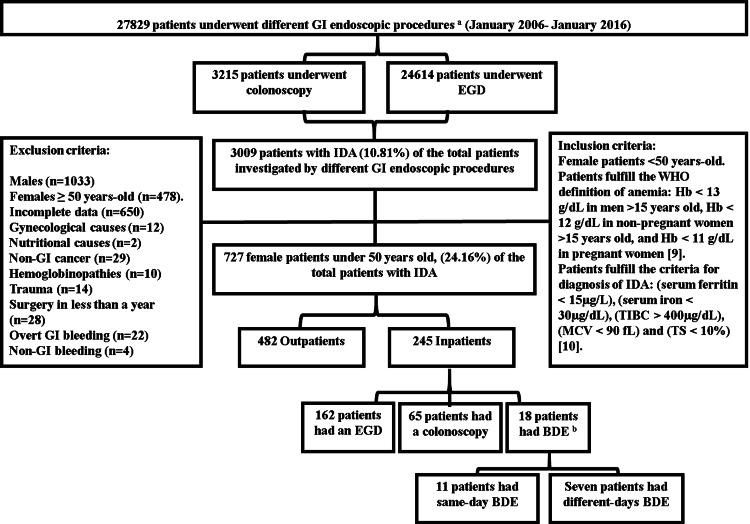
Flow chart of data collection during the study of 245 premenopausal women admitted for investigation of the cause of iron deficiency anemia. Abbreviations: BDE, bidirectional endoscopy; EGD, esophagogastroduodenoscopy; GI, gastrointestinal; Hb, hemoglobin; IDA, iron deficiency anemia; MCV, mean corpuscular volume; TIBC, total iron-binding capacity; TS, transferrin saturation; WHO, World Health Organization. ^a ^All GI endoscopic procedures utilized the Pentax Medical (HOYA Group, Shinjuku, Tokyo) or Olympus (Olympus Surgical Technologies America, Southborough, MA) endoscopic system. ^b^ When the EGD and colonoscopy were done in a period up to four weeks apart.

This observational study involved the retrospective evaluation of 245 PW with IDA under 50 years old who were admitted to the Al-Sadr Teaching Hospital, Faihaa Teaching Hospital, and Basrah Oncology and Hematology Center for management of IDA. The analysis involved medical paper data from January 2006 to January 2016 (i.e., before record automation). We evaluated data from endoscopy, laboratory, histopathology, and admission units of the three centers. Figure 1 illustrates the process of data collection, inclusion and exclusion criteria, and the selection of patients for final analyses. All the ultimately enrolled patients had measurements of their hemoglobin (Hb), iron status evaluation (serum ferritin and iron, total iron-binding capacity (TIBC), and transferrin saturation (TS)), and mean corpuscular volume (MCV) as a morphological parameter for the red blood corpuscles. Clinical and laboratory inclusion criteria are shown in the figures below, according to references [9,10].

The data were tabulated according to the individual demographic characteristics, initial symptomatology, duration of hospitalization, different GI endoscopic findings, and the severity of IDA. The parameters for severe IDA were Hb ˂9 g/L, serum ferritin ˂9 µg/L, MCV ˂70 fL, serum iron ˂15 µg/dL, TIBC ˃400 µg/dL, and TS ˂3.5 %, while for mild to moderate IDA they were Hb 9 to <13 g/L, serum ferritin 9-15 µg/L, MCV 70-90 fL, serum iron 15-30 µg/dL, TIBC 360-400 µg/dL, and TS 3.5-10% [10].

The analysis of GI symptoms included four categories. First, upper GI symptoms (dysphagia, indigestion, heartburn, nausea with or without vomiting, vague epigastric pain). Second, lower GI symptoms (any change in bowel habits, diarrhea or constipation, lower abdominal colic). Third, asymptomatic patients without any GI symptoms. Fourth, patients with mixed upper and lower GI symptoms.

The patients were diagnosed with a biopsy-proven CD if they have an endoscopic biopsy that revealed Marsh type 3 (A, B, and C), with different degrees of villous atrophy, according to the American College of Gastroenterology clinical guidelines [11]. The estimation of the anti-tissue transglutaminase IgA (ATTGA) and IgG (ATTGG) was done using enzyme-linked immunosorbent assay (ELISA) by BioTek ELx800 ((BioTek Instruments Winooski, VT, USA), and Alegria® by ORGENTEC Diagnostika (Mainz, Germany). A value >10 U/mL for ATTGA and >9 U/mL for ATTGG was considered positive. A value <4 U/mL for ATTGA and <6 U/mL for ATTGG was considered negative. The values between the positive and negative results were considered weak positive.

The Arab Board of Medical Specialization- Iraq gave ethical approval for the study. The retrospective design of the study showed no personal data for any of the enrolled patients who consented to different investigative procedures at the time of their initial evaluation (2006-2016). 

The data were entered and matched via Microsoft Access and Excel and then analyzed on SPSS Statistics for Windows, version 26.0 (IBM Corp., Armonk, NY, USA). We used the mean and the standard deviation to express the continuous variables, and percentages to express the categorical variables. General univariate analysis was used variables adjustment for the symptomatology as a dependent parameter. The study considered the two-tailed p-value ≤0.05 to be statistically significant.

## Results

The general characteristics of the cohort are expressed in Table 1. The mean age of the PW with IDA was in the middle age group (40±7) years, median 43 years. The mean duration of hospital stay was 2±1 days, with a range of one to eight days.

**Table 1:** The demographic characteristics of 245 premenopausal inpatients with IDA who underwent different GI endoscopic modalities. Abbreviations: BDE, bidirectional endoscopy; EGD, esophagogastroduodenoscopy; GIT, gastrointestinal tract, Ig, immunoglobulin; MCV, mean cell volume; SD, standard deviation; TIBC, total iron-binding capacity. ^a^ All the biopsy-proven CD were Marsh IIIA (eight cases), IIIB, and IIIC (four cases each). ^b^ BDE was the diagnostic approach for one upper, and two cases of lower GI malignancies. ^c ^There were no patients with serum ferritin ≤ 9 µg/L, serum iron <15 µg/dL, or transferrin saturation >3.5%.

Parameters		Value
Mean age in years (SD) (median)		40.44 (6.77) (43)
Mean age years for BDE in years (SD) (median)		37.61 (4.10) (36.5)
Mean age for women who had a colonoscopy in years (SD) (median)		43.74 (5.26) (45)
Mean Age for women who had EGD in years (SD) (median)		39.43 (7.10) (42)
Mean duration of hospitalization in days (SD)		2.42 (1.34)
Symptomatology	Upper GI symptoms (%)	85 (34.7)
	Lower GI symptoms (%)	61 (24.9)
	Asymptomatic (%)	71 (29)
	Mixed symptoms (%)	28 (11.4)
Celiac disease diagnosis	Referred for celiac disease (%)	44 (18)
	Anti-tissue transglutaminase antibody IgA (%)	21 (8.6)
	Anti-tissue transglutaminase antibody IgG (%)	20 (8.2)
	Biopsy-proven celiac disease (%) ^a^	16 (6.5)
Malignancies ^b^	Upper GI malignancies (%)	23 (9.4)
	Lower GI malignancies (%)	18 (7.3)
Iron deficiency^ c^ anemia parameters	Mean ferritin µg/L	13.09 (1.34)
	Mean hemoglobin g/L	9.31 (2.02)
	Mean corpuscular volume fL	73.18 (11.03)
	Mean serum iron µg/dL^3^	24.65 (4.08)
	Mean total iron-binding capacity µg/dL	379.24 (28.58)
	Mean transferrin saturation %^3^	6.53 (1.16)

**Figure 2:**
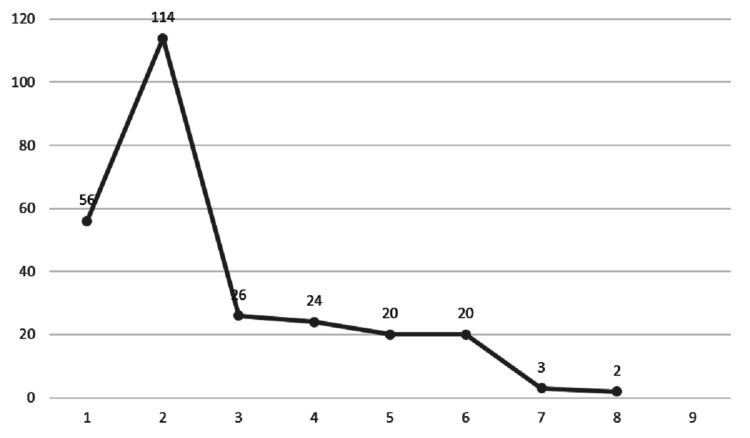
The duration of hospitalization of 245 premenopausal females who underwent different endoscopic procedures for diagnosis of iron deficiency anemia etiology. There were no associations between the length of hospital stay with either the age or the different iron deficiency anemia parameters, with P >0.05 by bivariate correlation analysis.

The IDA was of mild to moderate severity. The duration of hospitalization had no significant association with patients’ age or the severity of anemia (Figure 2).

**Figure 3:**
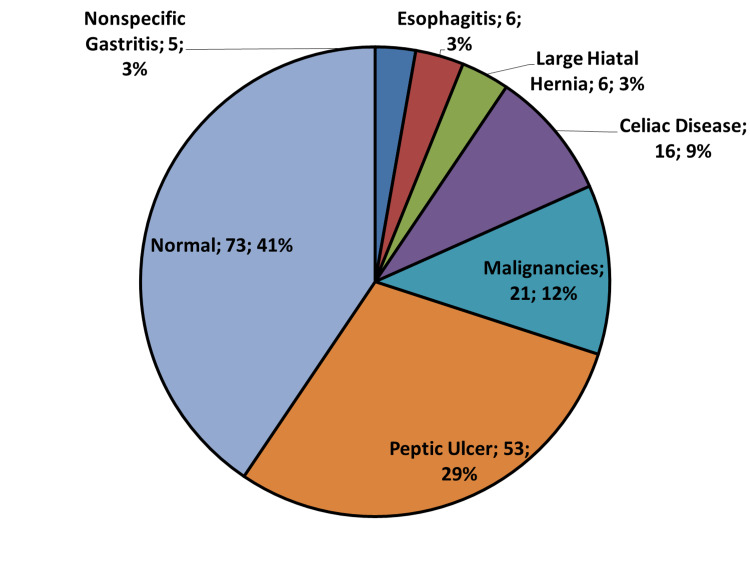
Pie chart of the significant upper gastrointestinal endoscopic findings of 180 premenopausal women with iron deficiency anemia. The results of the 18 premenopausal women who underwent bidirectional endoscopy were included in both panels. Abbreviation: GI, gastrointestinal.

**Figure 4:**
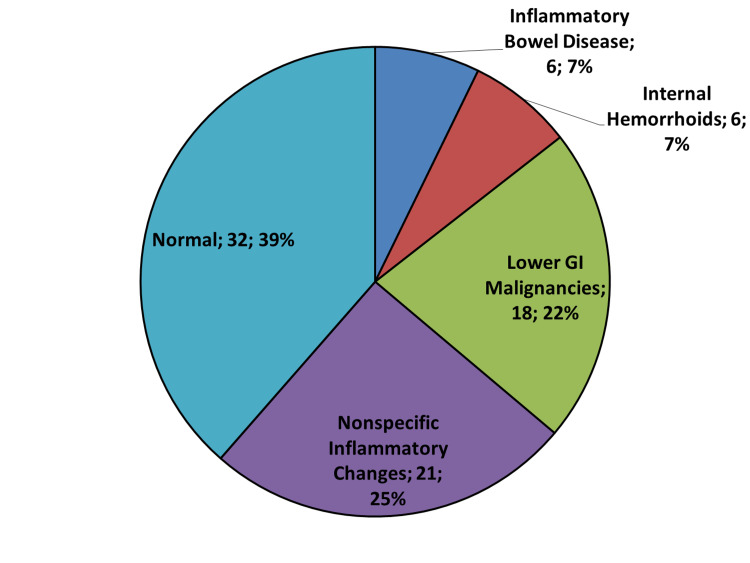
Pie chart of the significant colonoscopic findings of 83 premenopausal women with iron deficiency anemia. The results of the 18 premenopausal women who underwent bidirectional endoscopy were included in both panels. Abbreviation: GI, gastrointestinal.

Around 41% of patients (n=73) who underwent esophagogastroduodenoscopy (EGD) had a normal endoscopic study. Similarly, 39% who underwent colonoscopy (n=32) also had a normal study. Other findings in both EGD and colonoscopy were considered significant after exclusion of both normal and nonspecific inflammatory changes (Figures 3, 4).

There was a significant association between the initial GI symptomatology to both the significant GI finding and the diagnosis of GI malignancy (Table 2).

**Table 2:** The association between the symptomatology and the diagnostic outcome of 245 premenopausal women with iron deficiency anemia. Abbreviations: GI, gastrointestinal; MCV, mean corpuscular volume; TIBC, total iron-binding capacity. ^a ^The normal EGD studies and the nonspecific gastritis are excluded. ^b^ The normal colonoscopic studies and the nonspecific inflammatory changes are excluded.

Parameters	Upper GI symptoms n=85 (%)	Lower GI symptoms n=61 (%)	Asymptomatic n=71 (%)	Mixed symptoms n=28 (%)	P
Significant upper GI findings (n=102)^a^	41 (48.2)	22 (36.1)	24 (33.8)	15 (53.6)	0.002
Significant colonoscopic findings (n=30)^b^	11 (12.9)	14 (23.0)	5 (7.04)	0	0.015
Malignancy diagnosis (n=41)	18 (21.2)	17 (27.9)	4 (5.6)	2 (7.1)	0.002
Biopsy-proven celiac disease (n=16)	3 (3.5)	5 (8.2)	7 (9.9)	1 (3.6)	0.359
Hemoglobin < 9 g/L (n=69)	24 (28.2)	19 (31.2)	18 (25.4)	8 (28.6)	0.908
MCV < 70 fL (n=72)	23 (27.1)	21 (34.4)	21 (29.6)	7 (25.0)	0.746
TIBC > 400 µg/dL (n=16)	6 (7.1)	5 (8.2)	4 (5.6)	1 (3.6)	0.847

Such a relationship was not confirmed with the diagnosis of biopsy-proven CD nor to any parameter of IDA. Further analysis using the general univariate analysis of the significant factors failed to establish any significant association or demonstrate any power for these associations (Table 3).

**Table 3:** The general univariate analysis of the significant diagnostic outcomes when symptomatology is the dependent parameter.

Parameters	F	P	Observed power
Significant esophagogastroduodenoscopic findings	1.131	0.379	0.227
Significant colonoscopic findings	0.088	0.772	0.059
Malignancy diagnosis	0.303	0.593	0.080

## Discussion

Anemia is not uncommon and represents a unique challenge in PW. There are sparse data on IDA in PW because these patients are rarely referred for GI investigations [12], and there is no agreed-on consensus for endoscopic evaluation for asymptomatic PW [13,14]. Sporadic studies recommended bidirectional endoscopy (BDE) in the course of IDA evaluation of under-40-year-old PWs with IDA [8,15,16] even though significant GI pathologies were detected in published studies, with a substantial proportion of women with different GI malignancies being as high as 6% [5,7,8,17].

Nahon et al. and Dahlerup et al. reached a conflicting result about the use of BDE in PW with IDA as to whether it is recommended [17] or not [3]. The age range of this cohort was in the range of many other regional and international studies [13,15,18].

The coexistence of menorrhagia and iron malabsorption due to GI causes like CD, atrophic gastritis, *Helicobacter pylori* infection, peptic ulcer, and malignancy has been described in many studies, with a given prevalence from 20-35% of PW with IDA [3,5,8,15,16,19,20].

The approximate duration of hospitalization for diagnosis of the IDA etiology was 2±1 days with no correlation to the age of the PW or the IDA severity. We found no study that evaluated such a relationship.

We found no upper GI cause in around 43% of PW who had EGD - 73 patients had a normal EGD, and five had nonspecific gastritis - and in 64% of women who had a colonoscopy as a diagnostic procedure to search for the IDA etiology (Figure 3). Previous studies suggested that although it is possible to find a GI pathology, the majority have no significant findings, given an EGD widely varied diagnostic yield from 7% up to 55% [5,18,21]. This difference can be attributed to the different demographic characteristics between the studies.

There were 15.9% (n=39) of women with an endoscopic diagnosis of GI malignancies (21 with upper GI malignancies, and 18 with lower GI malignancies). The high percentage of GI malignancy in this cohort might reflect an inherent referral bias in this retrospective study because all the enrolled patients were referred to have GI endoscopic evaluation for IDA. Studies suggested that women of reproductive age are more prone to have a late presentation of GI malignancies, with a prevalence of 6-14% [5,18]. Other studies considered GI malignancy as a negligible cause of IDA in PW [13,15,22,23]. Age >50 years is a risk factor for colorectal cancer, and the majority of cases are older than 50 years old [15]. This contradiction in results was reflected in the British guidelines which recommend the GI endoscopic evaluation only for anemic postmenopausal women and men older than 50 years old with IDA [1].

There was a suspicion of CD in 18% (n=44), with biopsy-proven CD in 6.5% of the cohort (n=16). This was strengthened by the strong positive serology for ATTGA with or without IgG. The symptomatology had no significant relationship with the CD diagnosis. The European guidelines recommend CD screening for all PW with IDA, side by side, with serological CD testing [1]. In patients with CD, there is a mismatch between the iron requirement and absorption [24]. The course is subtle or silent in most cases, with a prevalence from 1-17% [18,20,25]. Mahadev et al. recommended performing the upper GI endoscopic biopsy for patients with IDA with high suspicion of CD, irrespective of other possible etiologies and any patients’ demographics [26]. There was a considerable discrepancy between the CD prevalence reported in the present study and other worldwide studies, which could be attributed to individual population-specific prevalence.

GI endoscopic diagnosis is symptom-directed, in general. About 71% of women had different spectra of GI symptoms (n=174), while 29% lacked any GI symptoms (n=71). There was a conflicting opinion on whether the presenting GI symptoms could guide the endoscopic evaluation in PW with IDA [8,14,18]. The symptomatology might affect the chance of referral for evaluation; still, this is not the case in 29% of this cohort. The only way to confirm whether we do unnecessary GI endoscopy in asymptomatic patients is by quantification of the menstrual blood loss, which is not possible given the retrospective design of the study, and even in prospective studies is subjective to personal estimation.

On the initial evaluation of GI symptoms, we found significant associations between symptomatology and the significant site-specific GI lesions and malignancy diagnosis. However, it is controversial to rely on the subjective symptoms to direct the endoscopic evaluation and the possibility of weak localization of visceral stimuli. On further adjustment of the variables with the symptomatology as a dependent parameter, the power of this association was lost (Table 3). Many studies considered the presence of GI symptoms as an independent predictor for positive GI endoscopy, with high positive and negative predictive values, and no need to perform endoscopy in women who lack GI symptoms [5,8,14,15,17]. Vannella et al. studied the pattern of symptoms and found a statistical association with the location of the GI lesions. Still, no association was found between the severity of anemia and endoscopic yield [20]. Authors suggested the poor response to iron therapy [15,27], and any related digestive compliant as indications for GI evaluation in young PW with IDA [15].

Another conflicting finding is whether to consider IBD as a significant finding in the etiology of IDA or not [5,16,20]. We had six cases of IBD and IDA, representing 2.4% of the cohort. Additionally, we had 71 patients with different findings: peptic ulcer disease (PUD) (n=53), internal hemorrhoids (n=6), large hiatal hernia (HH) (n=6), and severe esophagitis (n=6). Some investigators consider lesions like hemorrhoids, HH, nonspecific gastritis, esophagitis, and gastric ulcer <1 cm as unlikely to be the source of IDA [5,23], while others include them in the etiology [19,28].

Dual simultaneous upper and lower GI lesions were not identified in this cohort. A single endoscopic study could reveal the GI cause directly on some occasions unless there is a high index of suspicion that the diagnosed GI lesion is not the causative lesion. Given the similar endoscopic yield between the upper and lower GI endoscopy in different studies, it hard to prioritize one over another. The initial symptoms-directed endoscopy was not sufficiently diagnostic in 7.4% of the cohort (n=18), which had bidirectional endoscopy at different periods from each other. 

This study had limitations. We do not have any data about the admission criteria for our patients, indications of GI endoscopy other than IDA, or about the number of pregnancies, menorrhagia episodes, quantification of menstrual blood loss, dietary history, or iron supplementation. Failure to generalize the results and the possible selection bias due to the study's design were other limitations.

## Conclusions

There was a considerable percentage of PW with IDA who had an endoscopic cause for the IDA determined during upper or lower GI endoscopy. IDA in PW may be hindered by the presence or lack of subjective GI symptoms; therefore, these must be determined fully and individually. The severity of IDA did not affect the pattern of GI symptoms nor the duration of hospitalization for which the endoscopic diagnosis is taken on. The location of the GI symptom was neither associated with the location of the pathologic GI lesions nor with the GI malignancy diagnosis. Given the important endoscopic findings in PW with IDA, the GI endoscopy should be thorough and performed according to specific criteria in future prospective studies.
